# Highly-coherent second-harmonic generation in a chip-scale source

**DOI:** 10.1038/s41377-023-01359-0

**Published:** 2024-01-17

**Authors:** Xuanyi Liu, Hongyan Fu

**Affiliations:** Tsinghua Shenzhen International Graduate School, Tsinghua University, Shenzhen, 518055 China

**Keywords:** Nonlinear optics, Photonic devices

## Abstract

A highly efficient second-harmonic source is integrated into a silicon nitride microring resonator, unlocking the potential for advanced chip-scale devices such as miniaturized atomic clocks and fully integrated self-referenced microcombs.

Second-harmonic generation (SHG) is a nonlinear optical process in which photons of one frequency are converted into photons with twice the frequency. This phenomenon occurs in certain materials that lack inversion symmetry within their crystal structure. In a material with noncentrosymmetric properties, the electric field of an incident photon can induce polarization in the material, leading to the emission of a photon at double the frequency through a second-order nonlinear optical process. The SHG process requires a high-intensity coherent source, a material with second-order nonlinearity (*χ*^(2)^), and precise engineering of phase-matching conditions. To simplify experimental setups and enable on-chip integration of frequency doubling, it is crucial to employ silicon nitride (Si_3_N_4_), which is well-established as an integrated photonics platform, compatible with silicon-based complementary metal-oxide semiconductor (CMOS) technology, ensuring scalability and co-integration with microelectronics.

Recent findings reveal that Si_3_N_4_ waveguides and resonators can acquire a photoinduced *χ*^(2)^ through the coherent photogalvanic effect. Si_3_N_4_ waveguides now feature efficient SHG through all-optically induced quasi-phase-matching (QPM), demonstrating a *χ*^(2)^ value around 0.3 pm/V. This implementation has achieved a remarkable maximum conversion efficiency of 0.05% W^−1^ ^1^. Dispersion-engineered SiN photonic waveguides offer a versatile foundation for *χ*^(2)^ nonlinear optics utilizing femtosecond pulses, facilitated by the formation of self-organized nonlinear gratings^2^. Moreover, Si_3_N_4_ microring resonators have achieved SHG with an exceptional conversion efficiency reaching (2500 ± 100)% W^−1^ by combining a photoinduced effective *χ*^(2)^ nonlinearity with resonant enhancement and perfect phase-matching^3^. The occurrence of all-optical poling (AOP) has been demonstrated to be independent of intermodal phase-matching constraints, facilitating a broadly tunable SHG^4^. These on-chip techniques represent a significant breakthrough in achieving efficient *χ*^(2)^ processes within silicon photonics.

In a recent publication in *Light: Science & Applications*, Marco Clementi et al. propose a compact, power-efficient, and scalable approach to realizing frequency doubling and dual-wavelength operation^5^. They introduce a standalone, highly-coherent, and efficient second-harmonic (SH) source integrated into a high-quality factor (Q) Si_3_N_4_ microresonator. In their demonstration, self-injection-locking (SIL) and photoinduced *χ*^(2)^ coexist in a Si_3_N_4_ microring resonator, realizing a highly efficient dual-wavelength source emitting highly-coherent light at both the fundamental (FH) and second-harmonic (SH) frequencies, as depicted in Fig. 1. An electrically pumped distributed feedback (DFB) laser is edge-coupled to the Si_3_N_4_ photonic chip.

**Fig. 1:**
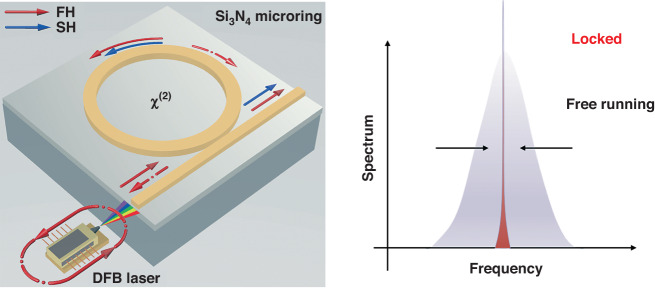
Schematic of the self-injection-locking (SIL) mechanism. The DFB laser injects light at the FH wavelength (solid red arrow) to the ring resonator bus waveguide. A small fraction of the light circulating inside the ring is reflected by Rayleigh backscattering (dashed arrows) and injected to the DFB cavity, yielding a dramatic narrowing of the emission linewidth compared to the free-running regime (right panel). Such high-coherence laser field displays a high intracavity intensity, which is used to trigger the coherent photogalvanic effect and generate SH light (blue arrow). Note that the backscattered SH light is not shown as it does not participate to the SIL process^5^

As illustrated in Fig. 1, the distributed feedback laser diode (DFB) directs light at the fundamental wavelength (indicated by the solid red arrow) into the bus waveguide of the ring resonator. A minor portion of the light circulating within the ring is reflected due to Rayleigh backscattering induced by sidewall roughness (illustrated by dashed arrows), and subsequently injected into the DFB cavity. Under appropriate phase and detuning conditions, the ring resonator functions as an efficient narrowband filter, causing the source to synchronize with its resonance frequency. This synchronization leads to a substantial decrease in the emission linewidth compared to the free-running regime, as illustrated in the right panel. The integration of DFB laser with the Si_3_N_4_ chip can be utilized to enhance the coherence properties, which is achieved through a self-injection-locking (SIL) mechanism to a microring resonator^6^.

The device exhibits remarkable performance characteristics, including a near-hertz intrinsic linewidth of 41 Hz, milliwatt-level SH output power, and an impressive side-mode suppression exceeding 60 dB. These features are maintained over a substantial locking bandwidth spanning several gigahertz, emphasizing the robustness and versatility of the integrated photonics chip. An innovative aspect of this device lies in its adaptability through the application of an AOP technique. By leveraging this method, the system can operate across the entire C and L telecom bands by solely in adjusting the sample temperature and pump wavelength, showing the efficiency and simplicity of the proposed approach.

The designed Si_3_N_4_ chip introduces an innovative method for on-chip SHG sources. The resonant element serves not only to boost the conversion efficiency but, more importantly, enhances the coherence properties of both the FH and SH fields through the SIL mechanism. The combination of SIL and AOP shows potential for extending to additional processes. For instance, the cascaded sum-frequency generation of the optical third-harmonic could be achieved through suitable optimizations of the source^7^. These optimizations may involve increasing the circulating power, engineering a triply-resonant condition, and enhancing light extraction at the third-harmonic wavelength. Moreover, through precise dispersion engineering, there is the potential to leverage the same microring resonator for the generation of a self-starting soliton microcomb^8^. As a noteworthy application, the frequency doubling of the microring resonator holds promise for on-chip *f*−2*f* interferometry^9^. This expanded functionality not only underscores the versatility of the microring resonator platform but also highlights the possibilities for diverse on-chip applications in precision metrology.
